# Developing an explanatory theoretical model for engagement with a web-based mental health platform: results of a mixed methods study

**DOI:** 10.1186/s12888-021-03391-z

**Published:** 2021-08-21

**Authors:** Dara Gordon, Jennifer Hensel, Zachary Bouck, Laura Desveaux, Charlene Soobiah, Marianne Saragosa, Lianne Jeffs, Sacha Bhatia, James Shaw

**Affiliations:** 1 Women’s College Hospital, Institute of Health System Solutions and Virtual Care, 76 Grenville St, Toronto, Ontario M5S 1B2 Canada; 2 University of Toronto, 155 College St, Toronto, Ontario M5T 1P8 Canada; 3 University of Manitoba, 66 Chancellors Cir, Winnipeg, Manitoba R3T 2N2 Canada; 4 St. Michael’s Hospital, 30 Bond St, Toronto, Ontario M5B 1W8 Canada; 5 Sinai Health System, 1 Bridgepoint Dr, Toronto, Ontario M4M 2B5 Canada

**Keywords:** Mental health, Virtual care, Depression, Anxiety, Digital health, Web-based, Mixed-method

## Abstract

**Background:**

With the growing need for accessible, high-quality mental health services, especially during the COVID-19 pandemic, there has been increasing development and uptake of web-based interventions in the form of self-directed mental health platforms. The Big White Wall (BWW) is a web-based platform for people experiencing mental illness and addiction that offers a range of evidence-based self-directed treatment strategies. Drawing on existing data from a large-scale evaluation of the implementation of BWW in Ontario, Canada (which involved a pragmatic randomized controlled trail with an embedded qualitative process evaluation), we sought to investigate the influences on the extent to which people engage with BWW.

**Methods:**

In this paper we drew on BWW trial participants’ usage data (number of logins) and the qualitative data from the process evaluation that explored participants’ experiences, engagement with and reactions to BWW.

**Results:**

Our results showed that there were highly complex relationships between the influences that contributed to the level of engagement with BWW intervention. We found that a) how people expected to benefit from using a platform like BWW was an important indicator of their future usage, b) moderate perceived symptoms were linked with higher engagement; whereas fewer actual depressive symptoms predicted use and anxiety had a positive linear relationship with usage, and that c) usage depended on positive early experiences with the platform.

**Conclusions:**

Our findings suggest that the nature of engagement with platforms such as BWW is not easily predicted. We propose a theoretical framework for explaining the level of user engagement with BWW that might also be generalizable to other similar platforms.

## Background

In 2016, more than one billion people worldwide were affected by mental illness and addiction [1]. Throughout COVID-19, national reports of mental health burden have increased tremendously, as a result of the psychosocial impacts of the pandemic [2, 3]. About 7% of the global burden of disease (as measured in disability-adjusted life years) and 19% of all years lived with disability were attributed to these disorders [1]. Ensuring the provision of high quality, affordable, and effective clinical services to meet the needs of this population remains difficult [4, 5].

In many jurisdictions with limited public funding for guideline-recommended psychotherapy, wait times are long and private services often require out-of-pocket payment and are unaffordable [5]. As a result, there has been increasing development and uptake of web-based mental health interventions, many of which are self-directed. During the pandemic, evidence shows that the use of these interventions increased drastically due to a wide-spread shut down of in-person services [6, 7] . This increase in web-based mental health interventions demonstrates their value for not just rural populations with limited access to conventional care as previously shown [8], but also urban populations across the globe [6, 7]. In this paper we define web-based mental health intervention as any interaction with a mental health care provider, peer, or information source focused on enhancing mental health that has been designed according to evidence-based standards for mental health and well-being.

Internet-based mental health interventions have been shown to be appealing, engaging, and efficacious in randomized controlled trials, especially for depression and anxiety [9, 10]. They offer strong potential to improve access to care as well as mental health outcomes, and can increase users’ sense of empowerment and perceived quality of life [9, 10]. However, research has shown that there are many factors that contribute to whether individuals initially access and engage with web-based interventions for health management more generally [11, 12], and researchers are only recently beginning to identify the influences on beneficial engagement with web-based strategies to enable self-management and the care of mental illness and other chronic conditions in sustainable ways [13–15]. In a scoping review by Ryan et al., 2018, the authors present several theoretical perspectives of adherence to web-based interventions. They highlight multiple important integrating factors related to adherence and engagement including environmental, technological and support variables, as well as individual user demographics and psychological characteristics, that must not be overlooked when characterizing adherence [16].

In a study exploring the factors that influence the decision to adopt and engage with a remote monitoring program for chronic medical conditions with or without connection to live service providers, Cook et al., 2016, wrote that the adoption of telehealth interventions is based on an acceptance of need, perceived benefits of technology and ‘ease of use’ [6]. In their study, they found that having a positive attitude and a perceived need that could be met by the service influenced the decision to adopt and engage. Building on this, other research has shown that perceived personal relevance of the intervention, peer and counsellor support and regular dialogue support and frequent program updates were associated with increased user adherence to web-based interventions for chronic conditions [16]. Further examining the influences on engagement with web-based health interventions, Wang et al. suggest that one of the strongest pillars of web-based health communities is social support [17]. They note that availability of both social and emotional support in these communities is important, along with the opportunity to provide social and emotional support to others. They highlight evidence that suggests that receiving emotional support is associated with longer stay in web-based health communities [17].

Effort to encourage engagement with web-based mental health interventions may confront unique challenges, unlike those described by other authors. While such interventions can overcome treatment seeking barriers such as stigma, availability [18] and geography, they also rely on self-motivation, a characteristic often negatively impacted by mental health challenges such as depression and anxiety. In a study about a web-based depression prevention tool, Zarski et al. note that high autonomy and flexibility of web-based interventions supports low-threshold access to treatment but that this places high self-regulatory demands on participants which may entice treatment cessation [19]. Interestingly, they also note that even highly motivated individuals struggle to engage in web-based interventions for depression. To explain engagement, Zarski et al. draw on the theoretical framework of the health action process approach to explain the gap between intention and behavior. The framework posits that there are two phases, the first being a motivational phase, in which the intention to adopt a health behavior is developed, followed by the volitional phase, in which behavior is planned, prepared, and executed [10]. Based on this framework, they deemed that motivational self-efficacy, which is defined as the belief in one’s ability to perform the targeted behavior, is regarded as the second best predictor of behavioral intentions. Zarski et al.’s findings also suggested that, individuals with high levels of maintenance self-efficacy may find it easier to engage in planning than those with low levels, as the former are confident that they can overcome adherence barriers such as technical problems and the absence of immediate feedback [10].

While this research begins to highlight trends that contribute to engagement with web-based mental health interventions, including expectation of what the intervention can offer as well as usability and an absence of technical issues, it is clear that there has been limited study and there is no strong consensus in the literature. In this paper we present a model that brings together both qualitative and quantitative findings from the evaluation of a multi-component web-based mental health intervention called Big White Wall (BWW). We present an additional schema of influences to provide insight into what drives engagement with web-based mental health interventions such as BWW, and discuss the role of this type of intervention within health systems.

## Methods

The analysis reported in this paper was part of a large-scale evaluation of BWW in Ontario, Canada that took place over 1.5 years starting in July 2016. The evaluation of BWW involved (1) a pragmatic randomized controlled trial and (2) a parallel qualitative process evaluation of the implementation of BWW to explore participants’ experiences and reactions to BWW in terms of their level of engagement and interest in continued use of the platform. Details and results for the randomized controlled trial have been published [20]. This paper draws on quantitative BWW usage data for the trial intervention participants and qualitative data from the process evaluation.

### BWW

BWW is a web-based platform for people with mental health challenges based in and operated from the United Kingdom. The application offers a range of components that incorporate evidence-based treatment strategies for mental well-being, all monitored continuously by “Wall Guides.” These guides are trained mental health professionals based in the United Kingdom who monitor for respectful and appropriate content and engage with users as required. These individuals review user activity and posts to ensure the content is appropriate and sensitive to all users. They will engage with users through real-time communication and where a user is in crisis, the Wall Guides can identify the location of the user and encourage them to use local crisis services. The Wall Guides do not have the ability to unmask the user’s identity. Additional details about BWW and its components, which are designed to be quite broad to support a variety of psychiatric disorders, are available in the published trial (Hensel et al., 2019) as well as the intervention website.

BWW contains a number of components, which are outlined in Table 1 below:

**Table 1:** BWW Components

Component	Description	Goal of Component
Useful Stuff Pages	Self-directed educational tools on various mental health topics structured in 4 stages of well-being: a) Self-assessment b) Understanding more c) Moving forward d) Building skills (examples include: depression, anxiety, trauma)	The Useful Stuff Pages provide information on mental health conditions and interventions.
Bricks	Artistic self-expression on a visual representation of a “brick” that becomes part of the community wall	The community wall is a space for users to post self-expression statements through the creation of artistic bricks.
Brick Comments	Comments posted by a user about one’s own brick or in response to another brick in the wall	Users can comment on each other’s bricks as a means to find community.
Guided Support Courses	Self-initiated with educational material and suggested activities	The Guided Support Courses are based on principles of cognitive behavioral therapy and behavior change.
Peer Community	User initiated discussions in the form of: a) Personal “talkabouts” (1:1 conversation with a Wall Guide or another BWW user) b) Community “talkabouts” (posting in a community conversation with other BWW users and Wall Guides) c) Group “talkabouts” (posts within groups of users taking the same guided support course)	Moderated and personal group “talkabouts” allow users to converse with wall guides and peers regarding their mental health concerns and experiences.

In this paper, we use the number of logins on the platform as a proxy for engagement. While engagement can be conceptualized across a spectrum of use metrics and immersion in content, number of logins is the most commonly cited metric in the literature [21, 22]. In contrast to metrics of adherence which often use percentage of assessments or modules completed, [9] BWW cannot be represented well in terms of modules since only one of its several components could be considered modular, i.e. the guided support courses (which are optional and self-initiated with educational material and suggested activities).

Study participants were recruited from three participating hospitals in Ontario and their affiliated programs. Participants were recruited by health care staff from a variety of settings including general psychiatry consultation clinics for adults and youth (primarily referrals for mood and anxiety disorders), a substance use program, crisis services (an emergency department and an urgent care clinic), a general psychotherapy clinic, and specialized therapy programs for posttraumatic stress and borderline personality disorder. Recruitment targeted individuals on waitlists for these programs or those discharged from crisis services. If interested when approached by clinic staff, the participant’s contact information was provided to the research team and a research assistant reached out by telephone or met in person to discuss further. This study was approved by each of the research ethics boards at the three participating hospitals.

### Quantitative design

Participants in the randomized controlled trial were randomized 2:1 to receive immediate access to BWW for 3 months or delayed access after a 3-month period while serving as a control group for comparison [20]. The study reported here only used data obtained for the participants who had immediate access to BWW (*N* = 542).

### BWW utilization variables

Over the 3 month period of access BWW, account activation, number of logins, and total time on the site was retrieved for each participant by the BWW administrators provided to the study investigators for analysis. Accounts were considered activated if the participant completed the registration, regardless of whether or not they actually used the site. A login consisted of some activity on the site in any of the BWW components, and total time on the site was recorded in minutes.

### Participant characteristics

All participant characteristic data were collected at baseline prior to randomization. These included participant sociodemographic data and mental health histories, as well as expectations about the intervention and symptoms. Demographics and mental health variables included age, gender, ethnicity (white vs non-white), relationship status (in a relationship vs not in a relationship), employment status (full-time or homemaker with young children, part-time or homemaker without young children, not working – retired or actively looking, and not working not looking for work), recruitment setting, age of first onset of mental health problems, and prior 3-month mental health contacts. The mental health contacts variable was derived from self-reported hospitalization days and ambulatory care visits (emergency department visits, primary and specialist care, and community mental health care visits). This variable was categorized as high, moderate or low to approximately reflect number of days in contact with services during the exposure period. High included a hospitalization more than 2 weeks in duration, attendance at a day program, or 12+ outpatient visits. Moderate was a hospitalization of 4 to 14 days, or 3–11 outpatient visits. Low was a hospitalization of 3 days or less, or fewer than 3 outpatient visits.

To assess belief in the intervention and expected benefit, participants were asked to rate their agreement with the statement, “Self-help tools including on-line services and books are helpful for people with mental health problems,” using a Likert scale with 4 responses from definitely agree to completely disagree, and to indicate their outcome expectancy by responding to item #4 from the *Credibility and Expectancy Questionnaire* [23], which has been shown to correlate positively with psychotherapy outcomes [24] . Response options for outcome expectancy were 10% increments from 0 to 100%, and were grouped into < 50, 50, > 50% for analysis based on the distribution of responses. Symptoms were assessed with the *Recovery Assessment Scale-revised (RAS-r)*, the *Patient Health Questionnaire-9 item (PHQ-9) for depression*, and the *Generalized Anxiety Disorder Questionnaire-7 item (GAD-7) for anxiety*. PHQ-9 and GAD-7 scores were categorized using established severity cut-offs [25, 26] rather than using raw scores because of a hypothesized non-linear relationship between severity of symptoms and use of BWW. The 5 sub-scales of the RAS-r were separately totaled: (1) personal confidence and hope, (2) willingness to ask for help, (3) goal and success orientation, (4) reliance on others, and (5) not dominated by symptoms [27].

### Quantitative analysis

Account activation, total BWW logins, total time on BWW, and all covariates were summarized descriptively. To quantitatively identify characteristics associated with BWW use, we regressed the total number of BWW logins during the 3 months (our proxy for engagement) on the participant characteristics (covariates) via negative binomial regression with a log link. All analyses were completed with SAS version 9.4.

### Qualitative design

For the qualitative process evaluation, we sought to understand the processes by which the technology was implemented and the ways in which participants interacted with the technology over time. We recruited participants purposively to ensure we had a balance of (a) different age groups, (b) different geographical locations, and (c) different recruitment settings [28, 29]. The purpose was to achieve a sample of participants that had variable experiences of mental health. Participants were recruited by relaying the qualitative sampling goals to the quantitative research coordinator, who then identified potential participants who had already indicated their willingness to participate in the qualitative component of the study. A qualitative team member then contacted each potential participant by phone. Participants were recruited until a diverse sample was obtained according to the above-stated criteria. Exclusion criteria for the qualitative interviews overlapped with that for the quantitative study, and research team members were prepared to end interviews if a participant was intoxicated or engaged in abusive behavior; no interested participants were excluded from the qualitative study.

Participants (*N* = 14) were interviewed at two time points, and where participants declined a second interview, a new participant was recruited with a similar demographic profile from the same site to participate in an interview in order to comment on their experience of continued engagement or disengagement with BWW over time. Participants were interviewed within the first 2 weeks of receiving access to the BWW, and again a second time between within 2 weeks of completing their 12-week experimental period. The goal of this timing for the interviews was to gain insights related to early experiences using the BWW and then related to overarching reflections on their experiences at the follow-up interviews.

### Qualitative analysis

Analysis of qualitative interview data was completed using semantic thematic analysis strategies as described by Braun and Clarke (2006). The initial phase included a basic, descriptive analysis [30] that sought to represent the surface meanings of participants’ accounts of their use and continued engagement or disengagement with BWW platform. After all interviews were transcribed and coded using a basic descriptive coding scheme (inductively generated), similar codes were grouped and semantic themes identified. These themes were then brought into analytic dialogue with the quantitative findings to generate a synthesis between the two data sources. We organize participant logins into three categories: those who were high (8+ logins), intermediate (2–7 logins), and low (fewer than 2 logins) engagers with BWW.

### Mixed methods analysis

**Fig. 1:**
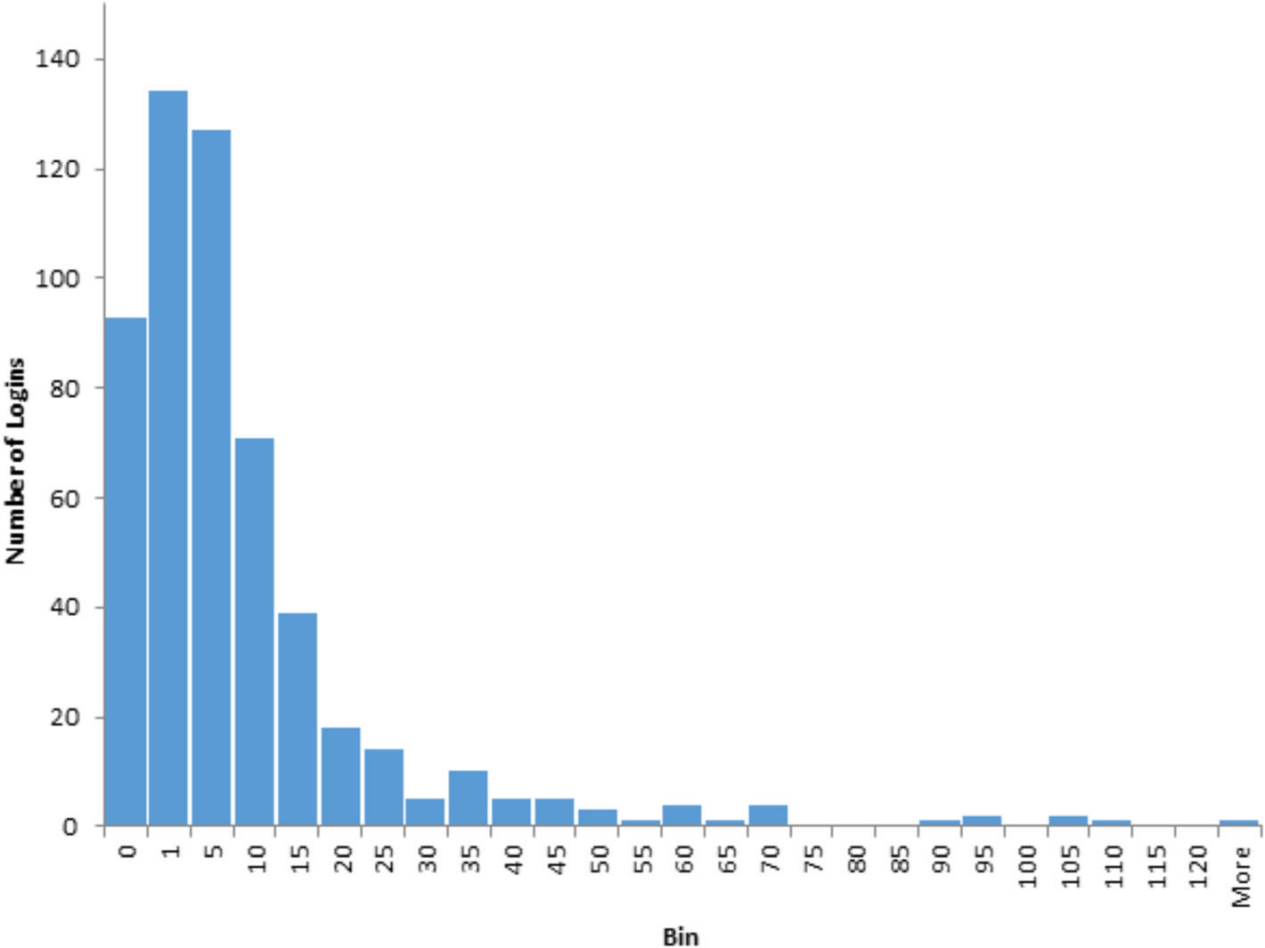
Frequency distribution of BWW logins among participants. Notes: Bin “0” = no logins (includes participants who did not activate their accounts (*n* = 76)); Bin “1” = 1 login; Bin “5” = 2–5 logins; Bin “10” = 6–10 logins, and so on. One participant in “More” at 236 logins

Our study followed a partially mixed concurrent, equal status design as described by Leech and Onwuegbuzie (2009). In such a design, the two components of the study (the qualitative and quantitative) take place independently and concurrently, with analyses brought together to generate insights that could not be obtained from either data source alone [31]. After qualitative data was analyzed thematically as described above, we qualitatively analyzed any relationships between qualitative themes and survey responses to the demographic and outcome measure surveys described earlier in the paper. We then compared qualitative interviews and survey responses with actual usage data from BWW, in order to more comprehensively categorize high engagers (8+ logins), intermediate engagers (2–7 logins), and low engagers (fewer than 2 logins). These categories were established based on the distribution of logins across the entire sample of participants, which was highly skewed (described in the results section). Given that we relied on number of logins as a proxy for engagement (a practice that is aligned with literature in this area), we deemed these categories to be the most appropriate cut-offs for our combined analysis. This analytic process was carried out over several meetings in which several data sources were discussed and the relationships between them critically analyzed. Between these meetings, team members leading the analysis (XX, XX, XX) wrote summaries and created data tables that could be shared with the team as a foundation for the analytic meetings. These categories formed the basis of our analysis and the development of our theoretical model presented in Fig. 1.

Paradigmatically we followed the insights described by Morgan (2007), wherein we relied on “abductive reasoning” [32] that moves back and forth between inductive and deductive analytic approaches to generate the theoretical model illustrating influences on engagement with BWW. The model is generated in equal parts by the qualitative and quantitative data, and the analytic work of the research team brought together insights from both independent data sources to build a model that is practically oriented.

## Results

### Quantitative data

The characteristics of the study participants who received immediate access to BWW are summarized in Table 2. The overall sample was predominantly Caucasian female, in the early 40’s, with mental health problems since late adolescence, and the highest proportion recruited from general adult psychiatry clinics. Severe levels of anxiety and depression were reported by 38.2% (*n* = 207) and 30.6% (*n* = 166) of participants, respectively. Utilization of BWW was highly variable. Of the 542 participants, 466 (86.0%) activated their BWW account and 446 (82.3%) logged on at least once. Only 57.6% of participants (*n* = 312) used BWW two or more times. The distribution of logins was highly skewed as shown in Fig. 1, ranging from zero logins (*n* = 93) to 236 logins (*n* = 1) per individual. The mean number of logins was 8.7, with a standard deviation of 18.1, a median of 2, and a mode of 1. In fact, the commonly observed 80/20 rule for population-level effects [33] was approximately observed for BWW logins: 20% of participants (*n* = 109) logged on 3523 times, accounting for 75.6% of the 4658 total logins. Among users, the median total time on the site was 43.5 min (IQR 8.2 to 152.7). Total time was highly correlated with number of logins (R^2^ = 0.85).

**Table 2:** Sample covariates assessed at trial baseline (*N* = 542)

Categorical variables	N	%^**a**^
Recruitment Setting (missing = 20)		
Youth Clinics	27	5.2
Psychotherapy Clinic	29	5.6
Specialized Therapy Programs	77	14.8
Crisis Services	92	17.6
Substance use disorder program	100	19.2
General Psychiatry Clinics	197	37.7
Gender (missing = 4)		
Transgender	1	0.2
Woman	393	72.5
Man	144	26.6
Ethnicity (missing = 0)		
White	444	81.9
Non-white	98	18.1
Relationship Status (missing = 0)		
In a relationship	288	53.1
Not in a relationship	254	46.9
Employment Status (missing = 3)		
Full-time	181	33.6
Part-time	100	18.6
Not working - retired or actively looking for work	76	14.1
Not working – not looking for work	182	33.8
Agree with: Self-help tools helpful for people with mental health problems (missing = 0)		
Somewhat or definitely agree	518	95.6
Somewhat or completely disagree	24	4.4
How much expected improvement in mental health through BWW (missing = 0)		
Less than 50%	195	36.0
More than 50%	204	37.6
50%	143	26.4
Prior 3-month mental health contacts (missing = 9)		
High	173	32.5
Moderate	242	45.4
Low	118	22.1
PHQ-9 depression score (missing = 0)		
Severe	166	30.6
Moderate-severe	122	22.5
Moderate	125	23.1
Mild	90	16.6
Minimal	39	7.2
GAD-7 anxiety score (missing = 0)		
Severe	207	38.2
Moderate	70	24.5
Mild	133	12.9
None	132	24.4
**Continuous variables**	**Mean**	**SD**
Age (missing = 0)	41.1	13.4
Age first experienced mental health problems (missing = 0)	18.7	12.5
RAS-r subscales (missing = 3)		
Personal confidence and hope	27.0	6.7
Willingness to ask for help	10.8	2.7
Goal and success orientation	17.8	3.7
Reliance on others	15.3	2.9
Not dominated by symptoms	7.1	2.7

^a^Percentages are calculated after missing data removed

*PHQ-9* Patient Health Questionnaire-9 item for depression

*GAD-7* Generalized Anxiety Disorder Questionnaire-7 item for anxiety

*RAS-r* Recovery Assessment Scale-revised

Results of the regression analysis are reported in Table 3. Individuals from general adult psychiatry clinics, females, those out of work, and those with greater levels of anxiety as assessed with the GAD-7 had significantly more BWW logins than their demographic counterparts. All PHQ-9 severity categories had fewer logins than the minimal depression referent group, but the difference was significant only for the moderate-severe depression group.

**Table 3:** Results of regression analysis, where outcome was the number of BWW logins over the 3-month access period

Variable	RR	95% CI	***P***-value
Recruitment Setting			
General Youth Psychiatry Clinics	.66	.34, 1.29	.23
Psychotherapy Clinic	.53	.30, .94	.03
Specialized Therapy Programs	.52	.35, .79	.002
Crisis Services	.69	.47, 1.02	.06
Substance use disorder program	.85	.58, 1.24	.39
General Adult Psychiatry Clinics	Reference		
Gender			
Transgender	1.02	.07, 15.41	.99
Woman	1.56	1.15, 2.10	.004
Man	Reference		
Ethnicity			
White	1.31	.91, 1.89	.15
Non-white	Reference		
Relationship Status			
In a relationship	1.10	.83, 1.44	.50
Not in a relationship	Reference		
Employment Status			
Full-time	.79	.57, 1.10	.17
Part-time	.43	.29, .62	<.001
Not working - retired or actively looking for work	.60	.40, .90	.014
Not working – not looking for work	Reference		
Agree with: Self-help tools helpful for people with mental health problems			
Somewhat or definitely agree	.82	.45, 1.49	0.51
Somewhat or completely disagree	Reference		
How much expected improvement in mental health through BWW			
Less than 50%	.74	.53, 1.03	.08
More than 50%	.97	.70, 1.35	.87
50%	Reference		
Prior 3-month mental health contacts			
High	1.18	.80, 1.75	.40
Moderate	.88	.62, 1.23	.44
Low	Reference		
PHQ-9 depression score			
Severe	.57	.27, 1.20	.14
Moderate-severe	.47	.23, .95	.04
Moderate	.69	.36, 1.31	.26
Mild	.91	.49, 1.70	.76
Minimal	Reference		
GAD-7 anxiety score			
Severe	2.53	1.42, 4.51	.002
Moderate	1.91	1.09, 3.34	.02
Mild	1.48	.89, 2.45	.13
None	Reference		
Age	1.01	1.00, 1.02	.09
Age first experienced mental health problems	.99	.98, 1.00	.10
RAS-r subscales			
Personal confidence and hope, per 1-unit increase	.97	.94, 1.01	.10
Willingness to ask for help, per 1-unit increase	.96	.91, 1.01	.10
Goal and success orientation, per 1-unit increase	.97	.92, 1.02	.18
Reliance on others, per 1-unit increase	1.04	.99, 1.10	.10
Not dominated by symptoms, per 1-unit increase	1.03	.96, 1.10	.44

*PHQ-9* Patient Health Questionnaire-9 item for depression

*GAD-7* Generalized Anxiety Disorder Questionnaire-7 item for anxiety

*RAS-r* Recovery Assessment Scale-revised

### Qualitative data

We completed qualitative interviews with a total of 14 participants. Of these 14 there were 11 participants interviewed within 2 weeks of gaining access to BWW; of this original 11, there were 5 follow up interviews completed (6 of the original 11 were lost to follow up and refused second interviews). Participants lost to follow up in the qualitative study were all in the intermediate and low engagement categories. Research team members were able to make contact with only 2 of the 6 who were lost to follow up, and they both indicated they were not interested in continuing with the study. In order to supplement our qualitative data at this second time point, we purposively sampled an additional 3 participants who had already had access to BWW for the 3 month intervention period and were also in low or intermediate engagement categories, bringing the total number of interviews to 19 and total number of participants to 14.

**Fig. 2:**
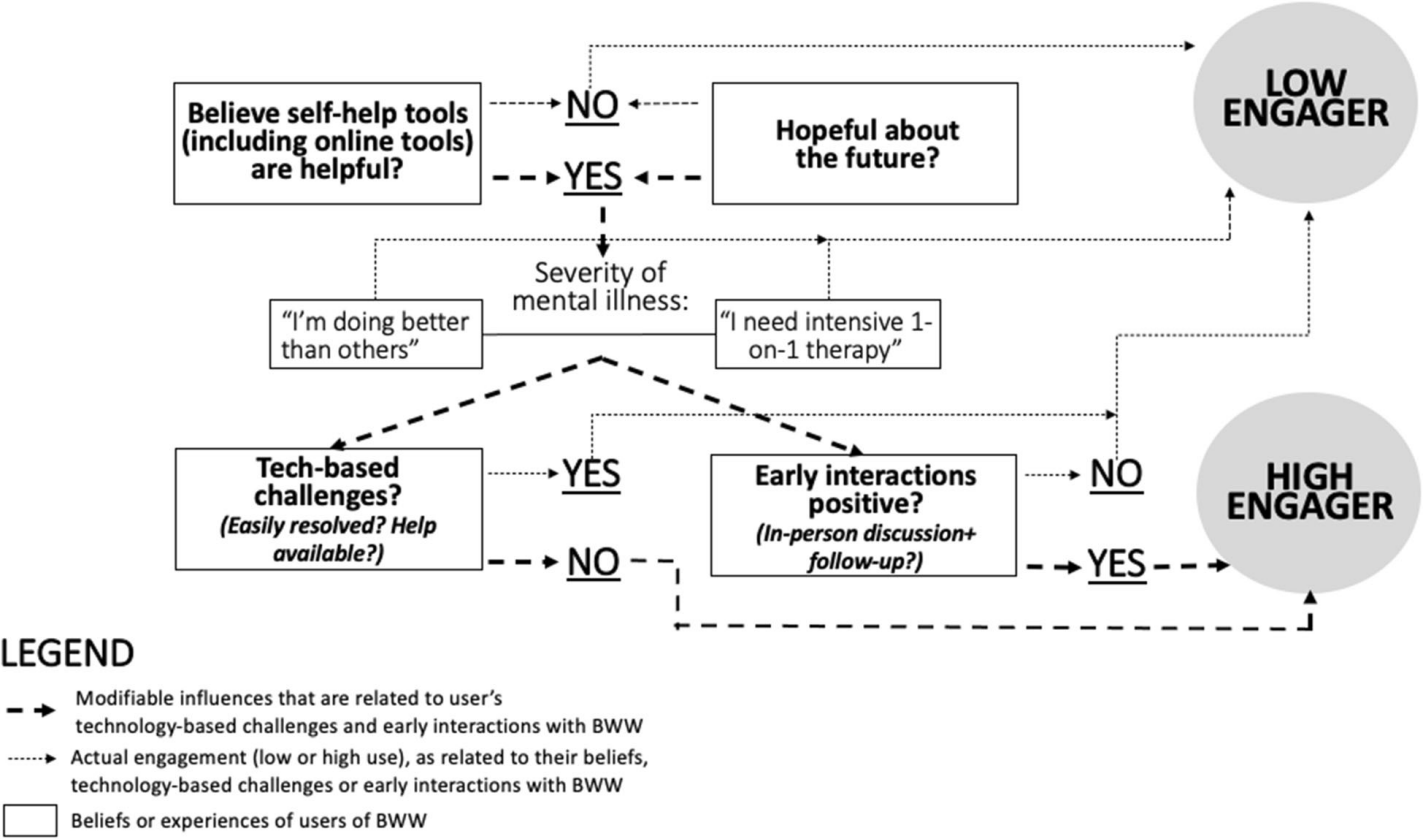
Determinants of Engagement

In this section we present an interpretation for the various levels of engagement that we observed. This is also depicted in a flowchart (Fig. 2) that illustrates the influences of the characteristics and user experiences that were associated with engagement or non-engagement with BWW during the early stages of BWW adoption. It is important to note that the data presented below represent an imperfect classification system; participants did not provide a homogenous picture related to a strongly coherent set of participant characteristics that clearly relate to higher likelihood of engaging in BWW. However, we suggest that the model generated by the insights in our mixed methods study represents a meaningful theoretical contribution to the literature on engagement in web-based mental health interventions.

While individual influences are subject to change over time, we found that when a particular set of influences occurred in confluence (or interacted together), trends related to engagement with BWW emerged. These 4 influences are described below. In this section, we report the characteristics of participants who were high (8+ logins), intermediate (2–7 logins), and low (fewer than 2 logins) engagers with BWW. The theoretical model we developed is depicted in Fig. 2.

#### Belief in self-help tools and hopefulness about the future

We begin by presenting two features of the survey data together (belief in self-help tools and hopefulness about the future); here we report the breakdown of responses to these two specific survey questions among participants in our qualitative study because we found that these two particular questions provided context for the narratives of our participants and the model we built in Fig. 2. We do not report these numerically, other than to identify how many of the participants we interviewed identified with particular survey responses (as such, this data belongs in the report of qualitative results, as opposed to the earlier report of quantitative results). For the first question, belief in self-help tools, all of those participants who were high engagers except for 1 (i.e., 6 out of 7 participants) reported believing that self-help tools (including web-based tools) were helpful (i.e., they responded “strongly agree” to the statement “Self-help tools including on-line services and books are helpful for people with mental health problems”). Intermediate engagers reported both “definitely agree” (*n* = 2) and “somewhat agree” (*n* = 3), and low engagers (n = 3) all reported “somewhat agree”. For the second feature, hopefulness about the future, high engagers (*n* = 7) all responded as “strongly agree” or “agree” to the statement, “I am hopeful about my future”. Intermediate engagers (*n* = 5) demonstrated a wider dispersion of views, with 3 participants reporting “agree” in response, 1 reporting “not sure”, and 1 reporting “disagree”. Low engagers also reported a variety of responses, with 1 reporting “strongly agree”, 1 reporting “disagree”, and 1 reporting “strongly disagree”. Overall, high engagers in our qualitative study demonstrated more optimistic responses to both the question pertaining to belief in self-help tools and hopefulness about the future.

#### Severity of mental illness

A second stage of adoption characteristics relates to participants’ beliefs about their mental illness severity, even for those participants who responded positively to questions about the benefits of self-help tools and being hopeful about the future. Those participants in our qualitative study who believed they had significantly *more* severe mental illness than other BWW users, and those participants who believed they had significantly *less* severe mental illness than other BWW users both generally ended up being low engagers with BWW. For example, one participant explained her lack of engagement with BWW as a result of her feeling that she needed more intensive in-person therapy.

*“Um, I think what would really help me um, would be access to at least weekly psychotherapy … 1 on 1 … Um, I’ve done quite a few groups and they are helpful, but in the end I need to work through quite a few things 1 on 1 with a therapist.” (P001).*

Some participants explained that after briefly exploring BWW, they felt that they were less distressed or better off than many of the other users encountered in the peer community. They felt that BWW was more appropriate for people who had more severe issues to address, and stopped using BWW as a result.

*“I was down and it wasn’t going so well and I wasn’t feeling optimistic and you know – actually in all fairness maybe the wall helped me to realize that I was actually doing better than I thought I was... Yeah, or the fact that I was able to, to see what it had, and participate in it, maybe just the participation in it you know, made me feel like hey, this is, ‘I’m good, I’m good to go, I’m better, I’m much better than I thought I was’.” (P002).*

This participant explained that perhaps it was his early experience with BWW that helped him to realize the extent of his own recovery, but nonetheless resulted in a clear tapering off of his use of the platform. In contrast to these participants’ narratives suggesting that they do not feel they are the ideal user of BWW, high engagers described being in an ideal phase of their recovery to engage.

*“There’s a lot of negativity – not negativity but just expressions that are you know hard to read sometimes, but I’m, I’m at a place in my recovery where I can deal with them, and [read] them and not take them to heart, where, I was talking to another person in day therapy and um … her son unfortunately committed suicide so her big trigger [pause], anytime somebody talks about it or mentions she shuts it down and she can’t do it.” (P003).*

#### Early experiences with BWW

In addition to participants’ beliefs about the severity of their mental illnesses, their early experiences with BWW were crucial in determining whether they would persevere and continue using the platform. This point is particularly challenging for proponents of the scale and spread of technologies such as this one, because it indicates that usage might be unpredictable from the outset of an implementation, scale, or spread initiative; adoption might depend on early experiences which cannot be known ahead of time. One participant explained how her early experiences on BWW made her feel unimportant.

*“I did post on there once during a really bad self destructive stage, where it had gone on for four days and it just got worse, so when I really did need immediate sort of um, talking to someone even with the wall guide, the response time just really sort of I guess you could say made me feel not important … Even though I know – it’s you know people can’t be online all the time, but that’s the sort of feeling that came across to me, its just like okay you know I’m in a crisis, I really, really need to talk to someone.” (P004).*

This participant acknowledged that the Wall Guides cannot possibly be monitoring every conversation all the time, but nonetheless felt neglected and ignored resulting in her feelings of rejection. Despite this participant reporting her dissatisfaction with this experience, she nonetheless used BWW frequently. This represents the *discordance between reports of satisfaction and actual usage*, signifying the immense challenge of predicting who will use web-based resources such as BWW.

This participant’s experience of early non-responsiveness from Wall Guides and other features of BWW is in stark contrast to other participants’ experiences. One participant explained his initial skepticism about the usefulness of the site, which was catalyzed into support when the community was extremely responsive for him during a time of crisis. The contrasting experiences between these two participants represents the unpredictability of an uncontrolled, public environment.

*“I had an episode I want to say last Sunday, or the Sunday – no two Sundays ago – my ex-husband actually contacted me for the first time in 3 years since the divorce. And I had a freak out, and it was in the middle of the night, he had contacted me through skype found my email somehow. And I had a – was shaken to the core, I went on the big white wall, and um, I typed like a mad fool about everything because I was in a total freak out and the wall guides were talking it through: “Remember what you’ve learned about in day therapy, the breathing, [etc.]” – it got me through the night … Yeah it was a major crisis, I was in total panic mode. I would have been so much worse if I didn’t have the big white wall.” (P005).*

In conventional therapy settings, an early connection with a therapist often helps to encourage ongoing engagement through the course of the therapy. However, like group therapy, the experiences of users of BWW will vary significantly depending on the nature of the other users and the specific Wall Guides with whom they are interacting at any given time. This unpredictability about the nature of the intervention is an important consideration in efforts to promote the widespread adoption of BWW, as it means that early experiences are modifiable but might influence whether users become high engagers or taper off their use in the early phase of adoption.

### Technology-related challenges in accessing BWW

An additional feature of the early experiences with BWW is the ease with which participants access the web-based platform. In this study, BWW was accessible only via web browser, not via mobile application. Accessing BWW in a web browser proved problematic for some people, with important implications for their engagement with the platform. One participant explained the challenges she had accessing BWW, leading to her abandonment of the technology over time despite early hope that it would work well for her.

*“[The project coordinator] emailed me through the links and unfortunately my email put it into junk and I didn’t get it until a day or two later, and she was like did you look at your junk and I did and there it was. As soon as I – and I logged in just by clicking on the link. And it looked fantastic and then that afternoon several hours later I had some free time it had notified me I had a new message and I had trouble getting back and I couldn’t access it through my cell phone, I forgot the username and password I had to set up. It did arbitrarily decide that my username I guess was too close to my real name so it assigned me a new one … Which is I understand why, but I was putting in the one that I first had – you know so I wrote down what I had used, but then as soon as I tried the one that they just assigned to me, I was able to log on, but not on my cell phone it just still won’t cooperate, I don’t know why … I just can’t it just kicks back “wrong user”, “wrong password”, and then it kicks back and it gives [United Kingdom/New Zealand] log in screen keeps popping up, which I don’t live there …*” *(P006).*

The Ontario-based users in this study had a unique landing page and login process for BWW, but the platform continued to be hosted in the UK and shared with existing users internationally. Although issues accessing the website might be expected when being introduced into a new jurisdiction, these features of the early experience were consequential for participants becoming high engagers or low engagers throughout the study period. Specifically, challenges in accessing technology was associated with lower engagement overall.

### How contributing factors interact to contribute to engagement or abandonment

Taken together, these characteristics of participants and their early experiences with BWW were important to understanding whether they became high, intermediate, or low engagers with the web-based community despite challenges to producing a clear, encompassing classificatory system.

In Fig. 2 below titled Determinants of Engagement, we provide a visualization of the determinants contributing to engagement with an accompanying legend. Depicted in the boxes are the beliefs or experiences of users of BWW. Depicted by thin arrows is their actual engagement (low or high use), as related to their beliefs, technology-based challenges or early interactions with BWW. Depicted by thicker arrows are modifiable influences that are related to users’ technology-based challenges and early interactions with BWW. The model begins with two particular questions related to a) belief in self-help tools being helpful and b) hope about the future. Regarding a) if users do not have belief in self-help tools (depicted by the arrow pointing to no), then they were found to be low engagers. If users do have belief in self-help tools (depicted by the arrow pointing to yes), then regardless of self-reported symptom severity, if they experienced tech-based challenges, despite them being easily resolved and help was available (depicted by the arrow pointing to yes), then they were found to be low engagers. If they did not have tech-based challenges (depicted by the arrow pointing to no), then they were found to be high engagers. Additionally, if users have positive early interactions with BWW, including in-person discussion and follow-up (depicted by the arrow pointing to yes), then they were found to be high engagers. If users did not have positive early interactions with BWW (depicted by the arrow pointing to no), then they were found to be low engagers.

Regarding b) if users are not hopeful about the future (depicted by the arrow pointing to no), then they were found to be low engagers. If users are hopeful about the future (depicted by the arrow pointing to yes), then regardless of self-reported symptom severity, if they experienced tech-based challenges despite them being easily resolved and help was available (depicted by the arrow pointing to yes), then they were found to be low engagers. If they did not have tech-based challenges (depicted by the arrow pointing to no), then they were found to be high engagers. If users are hopeful about the future (depicted by the arrow pointing to yes), then regardless of self-reported symptom severity, if they have positive early interactions with BWW, including in-person discussion and follow-up (depicted by the arrow pointing to yes), then they were found to be high engagers. If users did not have positive early interactions with BWW (depicted by the arrow pointing to no), then they were found to be low engagers.

Figure 2 presents an effort to construct a theoretical framework for better understanding the influences on whether and to what extent users engage with BWW web-based platform, but further work is required to refine when and how such influences operate.

## Discussion

In this mixed methods study examining influences for initial and sustained engagement with a multi-component web-based mental health application, we found that the characteristics of participants who were high, intermediate, and low engagers varied widely, both within and between these categories. A minority of participants were high engagers in general, with just over half of the sample using BWW two or more times. We found that most of the demographic factors did not contribute significantly to higher usage. However, we found that those who identified as female and those who were not employed and not looking for work were higher users. We suspect that we noted higher usage among females in part due to the fact that the study sample was 70% female owing to one site focussing on women’s health, and a general trend for females to have higher help seeking behaviour for mental health [34]. Similarly, higher usage rates for those who were unemployed may relate to this population having more time to access the platform, and possibly to a higher identified need as unemployment tends to correlate with illness severity [35].

### The complexity of influences on engagement

In order to interpret the model in Fig. 2, we first present three summative insights that we believe point toward the central implications of our findings, and then elaborate on those implications in the remainder of the discussion section with additional support from findings in the literature.

Our first major finding relates to believing in self-help tools and hopefulness about the future. Although not significant in the regression model, those with lower outcome expectancy did have fewer logins to BWW. Together these findings indicate that whether and how people expect to benefit from using a platform like BWW is an important indicator of their future usage.

Our second finding is about the relationship between mental health severity, perceived need, access to available treatment, and usage of the platform. Quantitatively, we observed fewer logins among those participants recruited from more intensive and specialized therapy programs. In the qualitative data we found that participants’ narratives about whether they used the platform were related to their perceived level of need and severity of their mental illness. Those who viewed their illness as more severe reported wanting more 1–1 intensive therapy and those who viewed their illness as less severe reported that this was not a contributor to their recovery or management. The quantitative findings point toward a more detailed understanding of the role of symptom severity in engagement with BWW. Specifically, those with more severe depressive symptoms engaged less, and more severe anxiety engaged more. This suggests that anxiety in particular may have a unique positive influence on engagement.

Our last finding relates to early experience and technology-based challenges on the platform. Our findings indicate that engagement is dependent on early experiences with the platform and therefore, a function of high engagement is actually a byproduct of usage itself. However, a user’s interaction with BWW is also unpredictable. They may have a positive or negative experience with the platform, they may receive helpful tech support that increases their engagement, they may develop a bond with a Wall Guide (Mohr et al., 2011 have written about human support in online systems), or they may feel a sense of community on BWW. These features are highly unpredictable, at least in the way that the intervention was managed and delivered in our study.

### Interpretation of the model

Our first major finding suggests that whether and how people expect to benefit from using a platform like BWW is an important indicator of their future usage. This is closely related to our third finding which suggests that usage depends on positive early experience with the platform (and likely other related technologies as well). These findings and the relationship between them are supported by a significant body of research on perceived usefulness, acceptability and ease of use of mobile mental health applications.

While research has highlighted that perceived helpfulness of an intervention is an important factor influencing the likelihood of use [36], much of this research suggests that knowledge of the application’s existence and potential benefit must precede any feelings of perceived or expected benefits. For example, in a paper on the acceptance of mobile mental health applications among German young adults, Becker noted that when users’ knowledge about the existence and effectiveness of the applications was low, expectations of their benefit and effectiveness were questioned and might have contributed to lower use (even in cases where the tools were considered easy to use) [37]. Gun and colleagues drew the same conclusion regarding web-based treatment for anxiety and depression; implementation and adoption of web-based mental health applications required strategies for increasing knowledge about the efficacy and effectiveness of the apps before users could perceive the apps as being useful or beneficial [38].

The literature also suggests that acceptance and perceived usefulness of web-based mental health interventions are mediated by previous personal experience [38]. For example, Gun et al. also reported in their work that former participants in web-based treatment for anxiety and depression reported higher acceptability after using the application and were more inclined to use web-based treatments in the future [38]. Mitchell et al. found a similar result with a web-based cognitive behavioral therapy (CBT) tool, wherein past learnings and early positive experiences promoted future intended use of the tool [39]. In conjunction with the literature just summarized, and building on our findings, this evidence indicates that promoting the benefits of web-based interventions for mental health to increase the public’s perception about their benefit may contribute to improved expectations and acceptability of these apps.

Our second major discussion point is related to self-reported symptom severity and most importantly the positive relationship between self-reported anxiety and BWW usage. Firstly, our findings that those with the most severe symptoms and perceptions of their needs reported believing they require specialized treatment resonates with findings from studies by Musiat et al. and Wootton et al., in which patients expressed that they thought their problems were too complex or severe to be treated online [36, 40]. Similarly, Becker found that while participants perceived mental health applications as being useful for the treatment of mental disorders, they did not think that mobile applications were, on their own, sufficient. However, in contrast to this observation in the literature, we found that moderate and higher levels of anxiety symptoms correlated with higher BWW use. In our trial, we did not find evidence that use of BWW was harmful [20], although this was not examined in detail, and is an important clinical consideration. Although we cannot provide any conclusive implications of this linear influence of anxiety on engagement, and this anxiety was based on self-report only rather than a clinical diagnosis, we suggest that further research should examine the relationship and implications between anxiety and engagement with web-based mental health interventions in greater detail.

### Health system & delivery implications

Our findings yielded several implications that relate to policies for procurement and provision of web-based mental health platforms. Assuming that an intervention is safe and inexpensive, then prescribing should ideally follow a population-wide approach that allows for self-selection into using the technology. Based on the 80/20 rule wherein a minority of the population will account for most of the usage [33], a user-based pricing model would not align with actual user engagement. If the price per user is high [41], then the therapeutic value or recommendations must be tailored such that they are most valuable to the people who are most likely to benefit for optimal cost recovery to the funder. Organizations procuring the technology may want to negotiate lower per user pricing, shorter user trial periods, or population-level pricing. In the case of user payment, clinicians must consider the financial status of the user, and the reality that certain lower-income populations may not be able to access it. A systematic review by Meurk et al. on establishing and governing e-mental health care in Australia suggests that research from a policy lens is needed to understand, “(1) the kinds of mechanisms available to government to facilitate implementation and (2) the imperative to fit e-mental health care within a population-based, stepped-care model that includes a range of treatment types for depressive and anxiety disorders and incorporates contingency planning” [42]. Although our findings provide support for a policy approach based on alternative funding models for technologies such as BWW, we suggest that further research is needed to establish the optimal policy framework for web-based mental health interventions more generally.

### Limitations

Despite the use of a strong study design, this study has a few limitations. Though the PHQ-9 and GAD-7 are validated tools across the spectrum of symptom severity, they are subjective such that self-assessment of severe anxiety disorders or depression could be distorted. No additional assessment was done because clinical assessments for participants would not have been feasible within the timeframe or budget of the trial. However, the aim of this study was to be very pragmatic; to understand the use and benefit of the intervention at a population level, and it was clear that there was no specific clinical recommendation or clinical follow-up related to use of the intervention.

Lastly, only 5 follow up interviews were completed from the original pool of 11 participants. To address this issue, additional participants who had already had access to BWW for the 3 month intervention period were purposively sampled (as described in the results section) to supplement our qualitative data at this second time point. Additional details related to the study drop outs are published in the main randomized control trial paper [20].

## Conclusion

Our study found that while the nature of engagement on a self-directed platform such as BWW is highly unpredictable, there were a few key factors that explained general patterns of engagement. These influences intersect in highly complex ways based on the nuanced needs of the users at specific points in time. This underscores the need to take a patient-centred approach to understanding how different people living with different, dynamic mental health challenges can benefit from digital health interventions such as BWW at different times in the course of their lives. Based on this type of varied engagement with platforms, brief subscription-based approaches to procurement of these interventions are not optimal, and therefore a self-selection model whereby users have some control over their usage is preferred. Additional research from a policy perspective that focuses on these specific needs is recommended to inform how these interventions could be adopted and sustained as a population health solution.

## Data Availability

The datasets used and/or analysed during the current study are available from the corresponding author on reasonable request.

## Abbreviations

BWW: Big White Wall
RAS-r: Recovery Assessment Scale-revised
PHQ-9: Patient Health Questionnaire-9
GAD-7: Generalized Anxiety Disorder Questionnaire-7
CBT: Cognitive behavioral therapy
